# Editorial: BODIPYs and Their Derivatives: The Past, Present and Future

**DOI:** 10.3389/fchem.2020.00290

**Published:** 2020-04-28

**Authors:** Hua Lu, Zhen Shen

**Affiliations:** ^1^Key Laboratory of Organosilicon Chemistry and Material Technology, Ministry of Education, and Key Laboratory of Organosilicon Material of Zhejiang Province, Hangzhou Normal University, Hangzhou, China; ^2^State Key Laboratory of Coordination Chemistry, Nanjing National Laboratory of Microstructures, Nanjing University, Nanjing, China

**Keywords:** BODIPY, dyes/pigments, spectroscopic properties, synthesis, borate complexes

Over the past decades, boron-dipyrromethene (4,4-difluoro-4-bora-3a,4a-diaza-s-indacene, BODIPY) fluorescent dyes, first described by Treibs and Kreuzer, have been the focus of considerable research interest and rapidly growing (Treibs and Kreuzer, 1968). Their structural versatility makes it possible to fine-tune their spectroscopic properties, and therefore they have been used in many scientific and technological fields (Loudet and Burgess, 2007; Ulrich et al., 2008; Boens et al., 2012, 2019; Kamkaew et al., 2013; Lu et al., 2014, 2016; Ni and Wu, 2014; Kowada et al., 2015; Zhao et al., 2015; Bañuelos, 2016; Sheng et al., 2019; Turksoy et al., 2019). This Research Topic mainly focuses on the most innovative research regarding the synthesis, spectroscopic properties, theoretical calculations, and application of BODIPY dyes and their derivatives. Four reviews and six original research articles by recognized academic experts are collected, which will offer a broad perspective for BODIPY chemistry and provide powerful guidance for the future rational design of BODIPY dyes and their derivatives with properties suitable for applications. We believe this Research Topic should attract the attention of multidisciplinary researchers and continue to promote BODIPY chemistry as a vibrant and highly multidisciplinary research field.

The contribution of Miao et al. summarizes fluorescent molecular rotors based on BODIPY for viscosity detection, providing key strategies for the design of various functional BODIPYs covering the red to NIR wavelength region for biological-related viscosity imaging. Triplet photosensitizers based on BODIPYs continue to attract increasing attention due to their extensive applications in photocatalysis, photodynamic therapy and photon upconversion. The contribution from Chen et al. reviews and classifies BODIPY-derived triplet photosensitizers based on ISC mechanisms, including the heavy atom effect, exciton coupling, and charge recombination (CR)-induced ISC, using a spin converter and radical enhanced ISC. Importantly, the molecular structure factors and mechanism of ISC-efficient are analyzed in-depth. This review affords fascinating insight for the rational design of novel BODIPY-based triplet photosensitizers. Typically, BODIPY dyes demonstrate weak fluorescence in the aggregation state duo to the self-absorption and strong intermolecular interactions, which restrict their application as solid-state emitters. In recent years, a number of AIE-active BODIPYs have been reported, but there remains a lack of general guidance regarding structural design. Therefore, Liu et al. summarize the AIE-active BODIPYs, their analogs boron-complexes, and their application in fluorescent imaging, gas sensors and as mechanofluorochromic (MFC) materials. The mechanism and structural factor for the aggregated fluorescent enhancement are further discussed to facilitate their future development. This review points out broad approaches for the design and application of BODIPYs as aggregation-state emitters, thus promoting, and enriching BODIPY chemistry. In addition to the three reviews already mentioned, which focus on molecular design and application, one contribution by Gupta and Kesavan concentrates on the synthesis and spectroscopic properties of BODIPY. Gupta and Kesavan summarize and classify BODIPYs containing a carbazole ring at *alpha, beta*, and *meso*-positions, and carbazole based hybrid BODIPYs, carbazole linked aza-BODIPYs, as well as carbazole-fused boron-complexes. The effects of a carbazole substituent in different positions on the optical properties of the BODIPYs are presented by tabulating their spectral properties.

The six research articles, on the other hand, focus on different aspects, mainly fluorescent probe and imaging, as well as synthesis and spectroscopic properties of bis-BODIPY and its optical limiting properties. Bartelmess et al. develop a BODIPY-cobaloxime complex for the detection of H_2_S in the liquid and gas phase. The selective substitution by the HS^−^ anion at the cobalt center releases the free BODIPY fluorophore, thus recovering the BODIPY fluorescence. The contribution by Wang et al. designs a FRET fluorescent probe for ratiometric detection of H_2_S *in vitro* and *in vivo*. Monochlorinated BODIPY can react with HS^−^ to form HS-BODIPY, thus affording a ratiometric fluorescent change. Interestingly, NIR-II fluorescence at 920 nm is observed, making the formation mechanism worthy of further study. The work by Qu et al. investigates a NIR BODIPY probe using triphenylphosphine as a reactive site for hydroxyl radical recognition and its bioimaging in HeLa cells, providing a new way to construct a molecular recognition system for biological application. Self-assembling BODIPY nanoparticles for bioimaging remain largely unexplored. The work of Ma et al. looks into the nanoparticles containing BODIPY with spherical and rod like morphology for cell imaging. Interestingly, the rod-like nanoparticles display great potential for bioimaging in efficient delivery and imaging efficacy, affording promising information for the design of bioimaging materials.

Oliden-Sánchez et al. describe the synthesis, photophysical, and lasing properties of a serial of bis-BODIPYs with spacers consisting of urea-, thiourea-, phosphonate-, amine-, disulfur-, and ether-based linkers. The spectroscopic behavior of bis-BODIPYs can be effectively tuned by the length and/or stereoelectronic properties of the spacer. The influence of the bridging moiety, solvent-effect and mechanism are systematically studied and analyzed in-depth, thus providing powerful guidelines for the future design of tailored bis-BODIPYs for wide applications. Non-linear optical properties of BODIPY have not been as extensively studied as their linear optical properties. The work of Ngoy et al. investigates the optical limiting properties of a 3.5-styryl BODIPY dye by using the open-aperture Z-scan technique. Since transparence of optical limiting materials remains a challenge, the authors point out how the structural modification of the BODIPYs to enhance this ESA, while shifting the main spectral band to the red, is the direction of future efforts.

## Author Contributions

All authors listed have made a substantial, direct and intellectual contribution to the work, and approved it for publication.

## Conflict of Interest

The authors declare that the research was conducted in the absence of any commercial or financial relationships that could be construed as a potential conflict of interest.
